# The feasibility of rapid baseline objective physical activity measurement in a natural experimental study of a commuting population

**DOI:** 10.1186/1471-2458-12-841

**Published:** 2012-10-04

**Authors:** Lin Yang, Simon Griffin, Cheryl Chapman, David Ogilvie

**Affiliations:** 1 MRC Epidemiology Unit, Institute of Metabolic Science, Box 285, Addenbrooke’s Hospital, Cambridge CB2 0QQ, United Kingdom; 2 UKCRC Centre for Diet and Activity Research (CEDAR), Institute of Public Health, Box 296, Forvie Site, Robinson Way, Cambridge CB2 0SR, United Kingdom

**Keywords:** Accelerometer, Natural experiment, Physical activity, Transport

## Abstract

**Background:**

Studies of the effects of environmental interventions on physical activity should include valid measures of physical activity before and after the intervention. Baseline data collection can be difficult when the timetable for introduction of an intervention is outside researchers’ control. This paper reports and reflects on the practical issues, challenges and results of rapid baseline objective physical activity measurement using accelerometers distributed by post in a natural experimental study.

**Methods:**

A sample of working adults enrolling for the Commuting and Health in Cambridge study and expressing willingness to wear an activity monitor was selected to undertake baseline accelerometer assessment. Each selected participant received a study pack by post containing the core study questionnaire and an accelerometer to wear for seven consecutive days, and was asked to return their accelerometer and completed questionnaire in person or by post using the prepaid special delivery envelope provided. If a pack was not returned within two weeks of issue, a reminder was sent to the participant. Each participant received up to five reminders by various methods including letter, email, telephone and letter sent by recorded delivery.

**Results:**

95% of participants registering for the study were willing in principle to undertake accelerometer assessment. Using a pool of 221 accelerometers, we achieved a total of 714 issues of accelerometers to participants during a six month period. 116 (16%) participants declined to use the accelerometer after receiving it. Three accelerometers failed, 45 (6% of 714) were lost and many were returned with insufficient data recorded, resulted in 109 (15%) participants re-wearing their accelerometer for a second week of measurement. 550 (77%) participants completed data collection, 478 (87% of 550) to the required standard. A total of 694 reminders were issued to retrieve unreturned accelerometers. More than 90% of accelerometers were retrieved after a maximum of two reminders.

**Conclusions:**

It is feasible to use accelerometers to collect baseline objective physical activity data by post from a large number of participants in a limited time period. However, a substantial pool of devices is required and researchers need to be prepared to make significant efforts to recover some of the devices.

## Background

Physical activity contributes to the prevention of numerous chronic diseases [1]. However, declining levels of physical activity have been reported in some developed countries despite considerable efforts to promote physical activity in different settings [2-4]. Environmental and policy interventions have been identified as the most promising strategies for achieving population-wide increases in physical activity [5]. Cross-sectional and longitudinal studies examining associations between characteristics of the built environment and levels of cycling or walking [6] suggest that improving transport infrastructure in ways that favour active travel may help influence people to take up cycling or walking instead of using cars, thereby increasing their overall physical activity. However, little evidence has been gathered from intervention studies in which the effect of infrastructural improvements on physical activity has been measured [7,8]; nor can such evidence easily be generated by researchers, who are rarely in a position to implement their own interventions in the built environment.

One way of addressing this lack of evidence is to conduct a natural experimental study to evaluate the effect of an intervention — in this case, a change to the environment involving improvements to transport infrastructure — that is not introduced for research purposes but is nonetheless amenable to evaluation [9]. Where such events occur that give rise to variation in exposure to interventions, researchers should consider taking the opportunity to evaluate their effects using robust, practical and cost-effective measures [5]. Guidance from the National Institute for Health and Clinical Excellence (NICE) [8] recommends that intervention studies of this kind should include valid measures of physical activity before and after the intervention to test associations between changes to the physical environment and changes in physical activity. In a natural experimental context, having no control over the implementation of an intervention sometimes constrains researchers to a limited time period for baseline data collection. In such circumstances, it is often easier to rely on the most commonly-used approach to measuring physical activity, which is to use self-reported measures [10]. However, it may sometimes be possible to incorporate objective measurement of baseline physical activity using devices such as accelerometers, even if the time available for data collection is limited.

A search of PubMed for studies using accelerometers to measure physical activity and published between 2005 and 2010 retrieved around 100 studies. More than half were cross-sectional studies, with sample sizes ranging from less than 100 to more than 2000. An example of a study at the upper end of this range is the Health Survey for England in 2008, in which 2115 adults were reported to have returned accelerometer data of a satisfactory standard for analysis [11]. Longitudinal studies have mostly been conducted in children, with studies such as ALSPAC [12][13] and SPEEDY [14] having collected accelerometer data from more than 1000 participants; however, certain longitudinal studies in adults such as the Nakanojo [15] and NHANES [16] studies have collected accelerometer data from more than 3000 participants, albeit not necessarily with more than one wave of accelerometer measurement. Relatively few intervention studies have been reported. The largest intervention study found in this search was that of a school-based intervention to reduce the prevalence of overweight in a sample of more than 3135 children and adolescents [17]. In this study, a total of 1538 participants from both intervention and control schools were randomly selected for objective physical activity measurement in weekly batches over a four-year intervention period. The largest intervention study among adults was a clinic-based behavioural intervention involving 236 women [18], of whom 178 were measured at baseline and followed up after six months and 173 were measured again after 12 months. The search found little evidence that objective physical activity measurement had been used in natural experimental studies, in which researchers have no control over the intervention. There is also little evidence-based guidance on how best to deploy accelerometers for the assessment of free-living physical activity in large studies [19].

A few studies have attempted to evaluate the effect of environmental interventions on active travel, but many have not included overall physical activity as an outcome [7] as recommended by NICE [8] and of those that have, few have incorporated objective measures of overall physical activity. For example, the RESIDE study has used survey and pedometer data to evaluate the impact of the Department of Planning’s Liveable Neighbourhood guidelines on the health and active travel of people moving into new homes in Western Australia [20], and in the UK the M74 [21,22] and iConnect [23] studies have used or adapted the short version of the International Physical Activity Questionnaire (IPAQ) [24] for baseline measurement, with iConnect including accelerometry only for specialist case studies. Studies of this kind sometimes encounter unexpected circumstances during the implementation of the intervention which require a high degree of flexibility on the part of researchers and, sometimes, of funding bodies. The challenges of completing baseline accelerometer measurement on a large scale in a limited time while maintaining a high level of data quality are therefore likely to be encountered by other researchers conducting similar studies in the future. In this study, we attempted the rapid collection of baseline accelerometer data from a large number of participants by post, without face-to-face contact. The aim of this paper is to report and reflect on the practical issues, challenges and results of this exercise in rapid baseline objective physical activity measurement in a natural experimental study.

## Methods

### Study design

In 2009, the Commuting and Health in Cambridge study was initiated in the city of Cambridge (UK) which had 108,863 inhabitants according to the 2001 Census [25]. The study design has been described in detail elsewhere [26]. In brief, it is a quasi-experimental cohort study designed to evaluate the impact of the Cambridgeshire Guided Busway on commuters’ travel behaviour, physical activity and related wider health outcomes. Three annual phases of data collection were planned. Baseline data collection involved a postal questionnaire for all participants coupled with objective physical activity measurement using accelerometers in a subsample. This was to be completed in 2009 before the opening of the busway. Ethical approval for the study was obtained from the Hertfordshire Research Ethics Committee and written informed consent was provided by each participant.

### Study population

Once ethical approval and other preparations were completed, recruitment began in March 2009 and six months were available for baseline data collection between May and October 2009. We recruited men and women who were over 16 years of age, travelled to work in Cambridge and lived within a radius of approximately 30 km from the city centre through a predominantly workplace-based recruitment strategy, using a range of methods including email, posters, leaflets and recruitment stands [26].

Commuters who were interested in taking part in the study were asked to register their interest, initially providing only basic data such as their gender, age group and home postcode and the area of Cambridge in which they worked. We used home postcodes and workplace locations to assess potential participants against the geographical inclusion criteria for the study [26], which required participants to live in a defined area and work in one of several areas of Cambridge that would be served by the busway. We began issuing survey packs to participants identified from the expression-of-interest database in weekly batches from the beginning of May 2009 and continued until the end of October.

### Willingness to participate

At registration, participants were also asked ‘As well as completing a questionnaire survey, would you be willing in principle to wear an activity monitor for a week?’ The intention was to issue accelerometers accompanying the core survey questionnaire to as many participants as possible who answered ‘Yes’ to this question. However, the number of accelerometer packs that could be issued in each batch was limited by the number of accelerometers available. As the study progressed, it became necessary to select a quasi-random sample of willing participants in each batch to receive an accelerometer and ‘roll over’ the remainder to subsequent batches of data collection. Towards the end of the data collection period, it had still not been possible to issue all willing participants with an accelerometer. Those who remained were therefore issued with a survey pack including only the core questionnaire.

### Data collection

We used Actigraph GT1M and GT3X accelerometers to assess physical activity. The Actigraph is a small, lightweight accelerometer that has been extensively validated for the assessment of physical activity in both laboratory and free-living conditions in different populations [19]. Participants received a survey pack containing an accelerometer, belt, instruction sheet and log sheet as well as the core survey questionnaire and consent form. They were asked to wear the accelerometer over the right hip using the elasticated waist belt provided during waking hours for seven days, removing it for bathing or swimming and logging any such removals. Upon completion, participants were asked to return their accelerometer and completed questionnaire in person or by post using the prepaid special (express, recorded) delivery envelope provided.

If an accelerometer pack was not returned, we sent a reminder letter to the participant two weeks after the issue date. If no response to the reminder letter was received, we then used a variety of approaches including sending further letters, sending emails or making telephone calls in each of the following weeks. As a last resort, final reminder letters were sent by recorded delivery. The maximum number of reminders sent to each participant was five.

## Results

### Study participants

Of 2163 people who registered their interest in taking part in the study, 2046 (95%) indicated their willingness in principle to wear an activity monitor and 1582 met the study inclusion criteria, of whom 714 were issued with accelerometers from the pool of 221 devices available during the baseline data collection period.

### Loss of participants and devices

**Figure 1:**
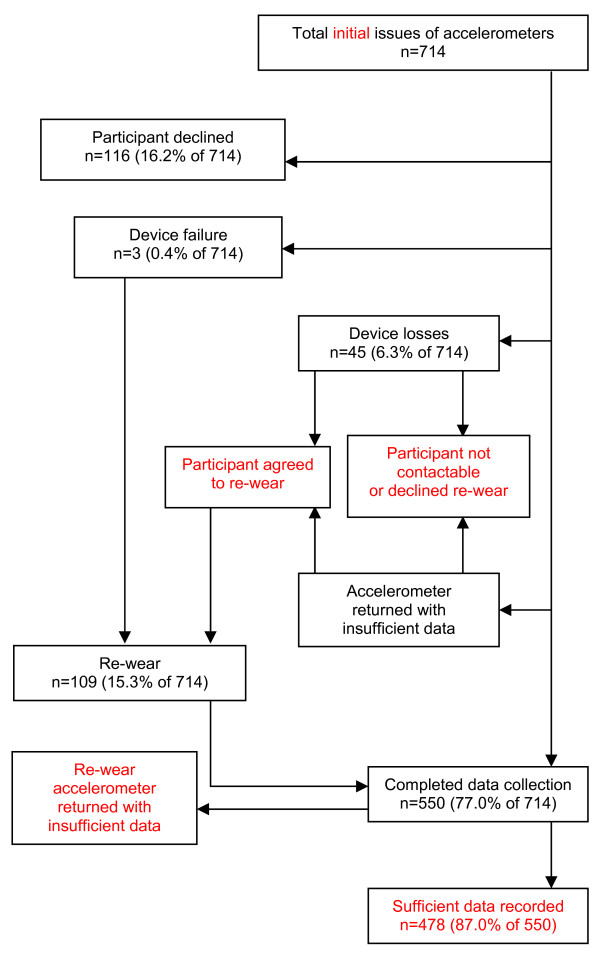
Flow chart for baseline accelerometer data collection in the Commuting and Health in Cambridge study.

116 (16.2% of 714) participants declined to participate in objective physical activity measurement upon receipt of their accelerometer (Figure 1). Three accelerometers failed (0.4% of 714 issues) and 45 (6.3% of 714 issues) were lost in distribution and return, which would cost approximately £9,000 (US$14,000) to replace. 35 devices were lost because participants did not respond to reminders and had provided no contact details other than their mailing address, nine were reported to have been lost in the post and one was mistakenly disposed of by a participant.

### Data quality

Of the accelerometers successfully returned to the study team, some had recorded insufficient data to meet the standard minimum requirement for analysis of 10 hours of data on each of four days [18,19]. Common reasons for insufficient data included participants having received their survey pack while on holiday or having been unaware of the need to begin using the accelerometer promptly because of its limited battery life. Unless participants had declined to participate or could not be contacted, we approached those affected by device failure, device loss or insufficient data to invite them to repeat their accelerometer data collection. 109 such re-wears were issued, accounting for 15.3% of the initial 714 issues of accelerometers and bringing the total number of accelerometer issues to 823. In the end 550 (77% of 714) of the participants originally issued with accelerometers returned any data, of whom 478 (87% of 550) returned data meeting the 4 days x 10 hours standard.

### Reminders

**Figure 2:**
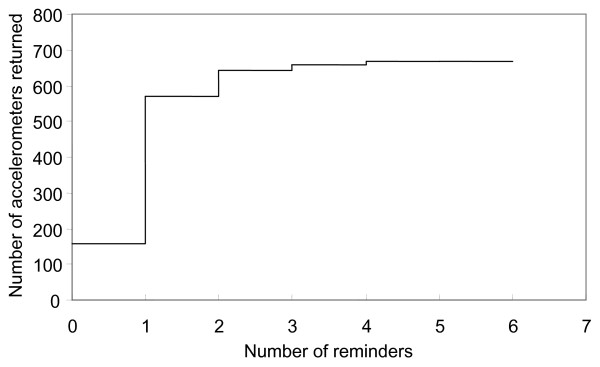
Accelerometers returned following each round of reminders.

Only 157 (22.0% of 714) of the original issues resulted in an accelerometer being returned with sufficient data within two weeks and therefore without any reminder being issued. A total of 694 reminders were issued. Only a few minutes were required to issue each reminder letter or email in the first and second rounds of reminders, by the end of which 644 accelerometers (90% of 714 issues) had been retrieved (Table 1, Figure 2). In a minority of cases more effort was required, for example involving telephone calls lasting up to 30 min in the fourth or fifth rounds.

**Table 1:** Average values of reminders issued to recover accelerometers

**Round**	**Number of reminders**	**Number of returns**	**Cumulative number of returns**	**Cumulative proportion recovered**	**Value of equipment recovered per reminder**
0		157	157	22.0%	
1	557	414	571	80.0%	£148.7
2	98	73	644	90.2%	£140.0
3	25	13	657	92.0%	£104.0
4	12	10	667	93.4%	£166.7
5	2	2	669	93.7%	£200.0

## Discussion

This study demonstrates the feasibility of rapidly assessing baseline physical activity in a natural experimental setting using accelerometers in a large sample of working adults. Our reflections on the practical issues and challenges encountered may help those intending to use similar methods in future studies.

### Implications

#### Willingness to participate

A surprisingly high proportion (95%) of those registering interest in our study expressed a willingness to wear an activity monitor. Although this suggests little difficulty in recruiting a sufficient number of participants, it may reflect an unusually high level of health consciousness in our relatively highly educated study population [27]: more than 70% of our study participants had a degree [28], compared with 40% of the population of Cambridge city and 18% of the population of England and Wales as a whole [29]. 16% of those who initially agreed to wear an accelerometer subsequently declined to do so after they received their survey pack. This probably reflects the fact that most participants were not recruited face-to-face; some may have underestimated the practical burden and inconvenience of wearing an accelerometer for seven consecutive days until they received the device and the accompanying instructions by post. The refusal rate in our study was higher than that in the intervention study conducted by Keyserling and colleagues, in which 10 out of 269 potential participants recruited declined to take part after attending their enrolment visit [18] and in the Health Survey for England [11]. On the other hand, the proportion of participants who eventually provided data of a satisfactory standard for analysis was higher in our study than in the Health Survey for England. It is important to anticipate likely refusals into consideration when planning the target sample size for recruitment, particularly if researchers plan to distribute study materials by post with no face-to-face contact with the participants.

#### Supply of accelerometers

While the accurate assessment of physical activity may be important for the quality of the research [5], using accelerometers is more expensive than using other commonly used methods such as questionnaires or pedometers [10] and it is unrealistic to expect that devices could be issued to every participant simultaneously when the sample size is large. Researchers therefore need to plan carefully for the recycling of devices during data collection to reach the target sample size, sometimes within a limited time period. We expected to recycle each accelerometer every four weeks: those four weeks would have included the time spent issuing and posting the survey pack, seven days of wear time, and the time spent returning the pack, downloading the data and re-initialising the device. As it turned out, we successfully issued accelerometers to 714 participants using a pool of 221 accelerometers, thereby issuing each accelerometer to just over three participants on average during the six month data collection period. This doubling of the recycling time reflects the additional delays caused by device loss, device failure and insufficient data recording and the resultant re-wears. It is important to be prepared to supply a sufficient number of devices to achieve the target sample size, especially when the time available to complete baseline data collection is constrained by the start of the intervention or other factors beyond the researchers’ control.

#### Administration and retrieval of accelerometers

In previous smaller studies, accelerometers have usually been distributed and collected through face-to-face meetings with a researcher. For larger studies, it may be impractical to arrange multiple individual meetings with each participant, especially when the time available for data collection is limited. One possible solution is to distribute accelerometers at face-to-face meetings and provide prepaid envelopes for their return by post [19]. This was the method used in the intervention study by Keyserling and colleagues [18]. However, our study involved a considerably larger sample and our initial contact with most participants was by email, so we had no face-to-face contact with most participants before they received their survey packs. We did provide padded packaging, prepaid special delivery envelopes and clear instructions to participants for returning their pack. Nevertheless, our experience of losing 45 devices in this study has several implications for the implementation of future studies.

Researchers should carefully consider the relative cost-effectiveness of alternative methods of distributing and retrieving devices, taking account of both postal charges and staff time. For example, recorded delivery — by which survey packs are tracked through the postal system from sender to recipient — may reduce the risk of losing devices in the post, but may be unsuitable in some studies because of the requirement for a signature upon receipt. We rejected this option for distributing our survey packs because our participants were commuters whom we expected to be out at work during the day when a recorded delivery might be made to their home.

Researchers should also emphasise to participants the importance of returning their accelerometer, especially if they decide they do not wish to take part. In the absence of a face-to-face briefing, the monetary value, use and care of the devices can be explained to participants before distribution using email. Advance email communication with participants can also help improve data quality and reduce the risk of re-wear by notifying the date on which the participant should expect their survey pack to arrive. Learning from experience in the initial batches of data collection, we altered our study procedures to include advance emails of this kind with more explicit instructions, for example emphasising the importance of commencing the seven day measurement period within two days of receipt because no more than nine days of battery life could be expected from the accelerometers.

In this study, over 90% of all accelerometer issues were retrieved with a maximum of two reminders using simple methods such as emails and ordinary letters. Including up to three further reminders using methods such as telephone calls and letters sent by recorded delivery increased the proportion of successful retrievals to 94%. Since each Actigraph cost approximately £200 ($320), the average value of equipment recovered as a result of each reminder was over £100 ($160) in each round (Table 1), even though the latter rounds of reminders recovered only a small proportion of the total number of devices. Our results therefore suggest that researchers should be prepared to issue multiple rounds of reminders to retrieve their devices and that doing so may remain cost-effective if the effort expended is gradually increased between rounds.

### Strengths and limitations

This paper contributes to the literature on the feasibility and practicalities of accelerometer data collection in population studies, specifically in the context of rapid baseline data collection in the natural experimental setting. A range of practical issues have been described and discussed with the aim of helping researchers who may wish to conduct similar studies in the future. Nevertheless, the characteristics of our relatively highly educated sample of commuters, our workplace-based recruitment strategies and our postal data collection protocol may limit the generalisability of our specific findings to studies with different characteristics.

## Conclusions

When conducting natural experimental studies, researchers should always be prepared for unexpected changes in circumstances that may require them to be flexible and adjust their study timetables and procedures. We have shown that it is feasible to use accelerometers to collect baseline objective physical activity data by post from a large number of participants in a limited time period. However, a substantial pool of devices is required and researchers need to be prepared to make significant efforts to recover some of the devices. Further economic evaluation of alternative data collection protocols would help guide the planning and allocation of resources for data collection in future studies.

## Competing interests

The authors declare that they have no competing interests.

## Authors' contributions

DO designed and led the Commuting and Health in Cambridge study in collaboration with SG (and others: see Acknowledgements). CC and LY collected the accelerometer data and collected and analysed the process data. LY and DO drafted the manuscript. All authors approved the final version.

## Pre-publication history

The pre-publication history for this paper can be accessed here:

http://www.biomedcentral.com/1471-2458/12/841/prepub
